# Propensity score-matched analysis comparing hippocampus-avoidance whole-brain radiotherapy plus simultaneous integrated boost with hippocampus‑avoidance whole-brain radiotherapy alone for multiple brain metastases-a retrospective study in multiple institutions

**DOI:** 10.1186/s12885-023-11286-3

**Published:** 2023-08-24

**Authors:** Xiaoliang Wang, Jinping Chen, Zhanquan Lei, Haihong Chen, Yufang Zhang, Gang Liu, Shaomin Li, Zhenhua Zheng, Hui Wang

**Affiliations:** 1 Department of Radiotherapy, The Third Hospital of Zhangzhou, Zhangzhou Fujian, 363005 China; 2 Department of Radiation Oncology, Army 73rd Group Military Hospital, Xiamen Fujian, 361003 China; 3 Department of Radiation Oncology, FuJian Children’s Hospital, Fuzhou Fujian, 350100 China; 4 Information Department, Army 73rd Group Military Hospital, Xiamen Fujian, 361003 China; 5 Department of Radiation Oncology, XiaMen ChangGung Hospital, Xiamen Fujian, 361028 China; 6 Medical Examination Center, Army 73rd Group Military Hospital, Xiamen Fujian, 361003 China; 7 Department of Radiation Oncology, Zhongshan Hospital Affiliated to Xiamen University, Xiamen, 361003 Fujian China

**Keywords:** Multiple brain metastases, Hippocampus-avoidance, HA-WBRT + SIB, HA-WBRT, Propensity score-matched analysis

## Abstract

**Background:**

The optimal treatment for multiple brain metastases has been recently controversially discussed.This study was aimed to explore the feasibility of Hippocampus-Avoidance Whole-Brain Radiotherapy plus a simultaneous integrated boost (HA-WBRT + SIB) in patients with multiple brain metastases and assess tumor control in comparison with Hippocampus-Avoidance Whole-Brain Radiotherapy (HA-WBRT) alone for brain metastases.

**Methods:**

In this study, 63 patients with multiple brain metastases (≥ 4 metastases) had undergone HA-WBRT + SIB between January 2016 and December 2020 in the observation group:HA-WBRT (30 Gy in 12 fractions, the maximum dose of the hippocampus ≤ 14 Gy) plus a simultaneous integrated boost (48 Gy in 12 fractions) for brain metastases.Overall Survival (OS), Median survival,intracranial control (IC = control within the entire brain), intracranial progression-free survival (iPFS) and adverse events were compared with the control group (a HA-WBRT retrospective cohort) by propensity score matching analysis.

**Results:**

After 1:1 propensity score matching,there were 56 patients in each group (the observation group, the control group). OS, median survival and iPFS were significantly longer in the observation group (18.4 vs. 10.9 months, *P*<0.001), (13.0 vs. 8.0 months, *P*<0.001), (13.9 vs.7.8 months, *P*<0.001). In comparison of 1-year-IC rates, the observation group also demonstrated higher than the control group (51.8% vs. 21.4%, *P* = 0.002), respectively. Seven hippocampal metastases were found in the control group (4/56,7.1%) and the observation group (3/56,5.4%) after HA-WBRT. The death rate of intracranial progression were 23.2% in the observation group and 37.5% in the control group.All adverse events were not significant difference between the two groups (*P*>0.05).

**Conclusions:**

HA-WBRT + SIB resulted in better OS,median survival, IC, iPFS, an acceptable risk of radiation response, and a potential way of declining neurocognitive adverse events, which may be a better treatment for patients with multiple brain metastases.

## Background

Brain metastases (BMs) are the most common intracranial tumors in adults. Brain metastases occur in 24–45% of all patients who are systemic tumors every year [1]. Among them, Brain metastases is common in lung, melanoma, breast, and renal cancer. Patients with one to three brain metastases usually were treated with Stereotactic Radiosurgery (SRS) or Stereotactic Body Radiation Therapy (SBRT) resulting in better local control (LC) and similar overall survival (OS). Although WBRT is a common treatment for multiple brain metastases, the best treatment is still controversial. Two retrospective studies demonstrated that WBRT plus SRS could improve local control but could not improve survival [2, 3]. In addition,132 patients did not achieve better survival through WBRT + SRS in a prospective trial [1]. In another retrospective study, patients without extracranial metastasis showed no better survival treated with WBRT + SRS compared to SRS alone [4]. However, the contradictory findings from the randomized clinical trials were that patients with one to three brain metastases were treated with stereotactic radiosurgery (SRS) [5]. Several studies have showed WBRT plus Simultaneous Integrated Boost (SIB) can improve overall survival of patients with brain metastases [6–8].

Neurocognitive decline was related to WBRT which led to hippocampal atrophy and long-term brain atrophy and leukoencephalopathy [9–11]. In addition, several studies showed that significant neurocognitive deterioration have also been related to poor IC and progressive brain metastases [12–14], and this demonstrated that IC is vital important to protect neurocognitive functions.

WBRT is associated with relatively low local tumor control (LTC),but better distant intracranial tumor control (DTC). On the contrary, SRS/SBRT displays higher LTC but relatively low DTC [15, 16]. Therefore, WBRT combined with SRS/SBRT can improve LTC of brain metastases [8]. Moreover, a phase 2 and a randomized phase 3 NRG Oncology CC001 trials demonstrated HA-WBRT preserved memory function [16, 17].

HA-WBRT plus SRS/SBRT could increase intracranial LTC,protect cognitive function,and also reduce neurologic death rates.

Prokic et al. [18] disclosed that The best way to protect the hippocampus can be attained if the dose escalation to brain metastases is planned as SIB instead of as sequential SRS in a planning study exploring the combination of HA-WBRT and SRS. Therefore, The purpose of this study was to explore the feasibility of HA-WBRT + SIB in the treatment of multiple brain metastases and assess OS, Median survival, IC, iPFS and adverse events in compared with HA-WBRT alone for brain metastases.

## Methods

### Patients’ selection

**Fig. 1:**
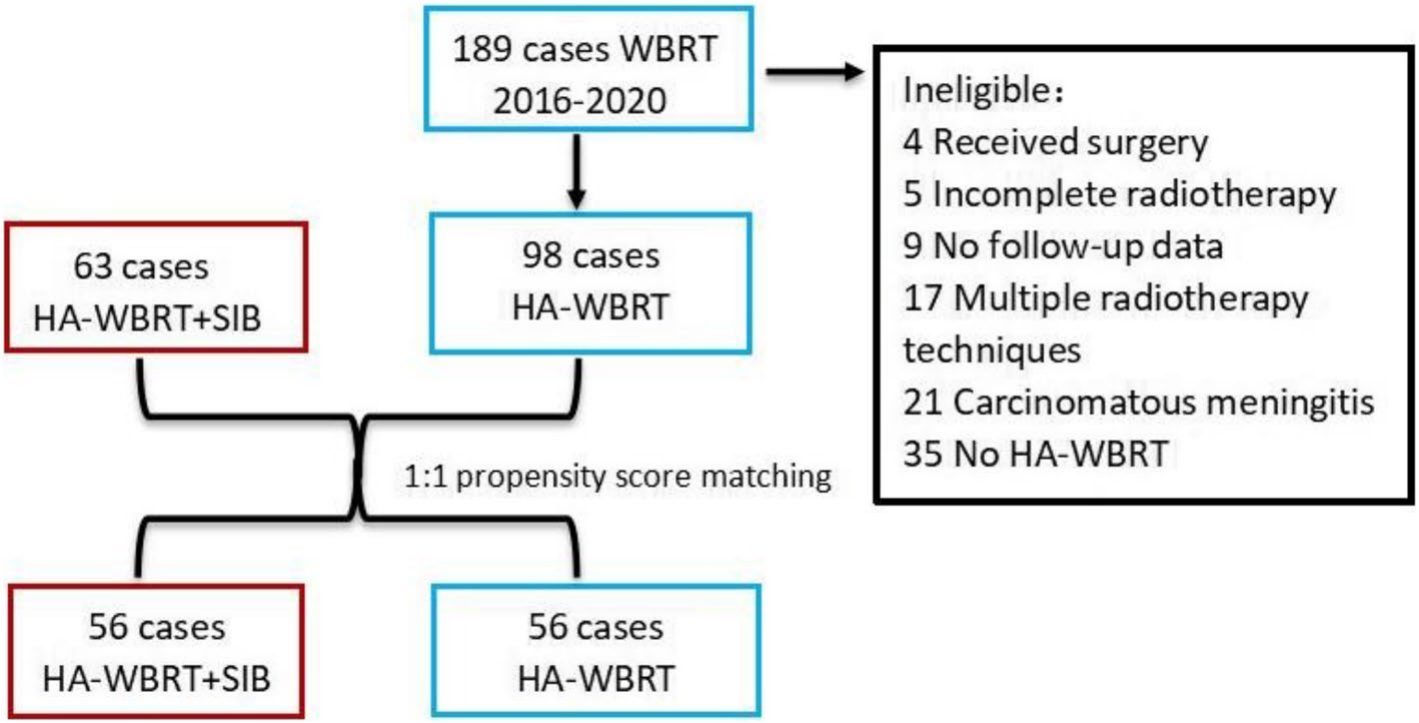
Eligible or ineligible for inclusion in the study. HA-WBRT + SIB indicates hippocampus-avoidance whole-brain radiation therapy plus a simultaneous integrated boost; HA-WBRT, hippocampus-avoidance whole-brain radiation therapy; WBRT,whole-brain radiation therapy

In this study, 63 patients with multiple brain metastases had undergone HA-WBRT + SIB between January 2016 and December 2020 at the Department of Radiation Oncology of the Third Hospital of Zhangzhou and XiaMen ChangGung Hospital in the observation group: Patients received HA-WBRT (30 Gy in 12 fractions, the maximum dose of the hippocampus ≤ 14 Gy) and a SIB (48 Gy in 12 fractions). The control group (a HA-WBRT retrospective cohort): 189 patients being retrospectively selected had been treated with WBRT between January 2016 and December 2020. Among them, 98 patients had received HA-WBRT and 91 patients were treated with WBRT without Hippocampus-Avoidance, who were not fit for further analysis. There were 56 patients in each group (HA-WBRT + SIB, *n* = 56; HA-WBRT, *n* = 56) through 1:1 propensity score matching (Fig. 1). There was significant difference in the small cell lung cancer (SCLC)(HA-WBRT + SIB, *n* = 7; HA-WBRT, *n* = 42, χ^2^ = 210.00, *P*<0.0001) therefore,patients with SCLC were excluded. Primary focuses of all patients were confirmed by pathology and enhanced-brain-MRI-proven multiple brain metastases (range 4–15), without hippocampal metastasis or 7 mm away from hippocampus. Those patients with one to three brain metastases and maximum diameter of BM less than 5 mm were excluded. Those patients who had no clear records at diagnosis or incomplete treatment were also removed. Baseline characteristics of patients was listed Table 1. The average characteristics of the patients include age, gender, karnofsky performance status (KPS score), number and maximum size of brain metastasis, brain edema, symptoms of BM, primary tumor, type of radiotherapy, extracranial metastases and systemic treatment.

**Table 1:** Baseline characteristics of patients

Patient Characteristic	HA-WBRT+SIB (*n*=56)	HA-WBRT (*n*=56)	*P* value
**Age(years)**, n (%)			0.295
**30~60**	23(41)	22(39)	
**60~80**	33(59)	34(61)	
**Gender**, n(%)			0.848
**Male**	33(59)	32(57)	
**Female**	23(41)	24(43)	
**KPS scores**, n(%)			0.894
**90**	13(23)	11(20)	
**80**	34(61)	36(64)	
**70**	9(16)	9(16)	
**Primary focus**, n(%)			0.194
**NSCLC**	39(69)	41(73)	
**Breast cancer**	6(10.5)	3(5)	
**Esophageal cancer**	6(10.5)	2(4)	
**Other cancer**	5(9)	10(18)	
**No. of BMs, mean(range)**, n	7.89 (4-15)	7.69 (4-15)	0.733
**Sum of maximum diameter of BMs mean (range), cm**	8.74 (3.5-19.1)	7.79 (3.4-19.7)	0.220
Volume PGTV, median (rang,cm^3^)	11.47 (5.35-29.21)	10.24 (5.2-30.13)	0.211
**maximum dose of the hippocampus median (rang, Gy)**	13.42(10.63-14.72)	12.85(11.2-14.12)	0.287
**mean dose of the hippocampus, mean (rang, Gy)**	7.84(5.56-9.35)	7.33(5.61-9.01)	0.071
extracerebral metastases, n(%)			0.552
Yes	18(32)	21(37)	
No	38(68)	35(63)	
Complications of BMs,n(%)			0.237
Yes	33(59)	39(70)	
No	23(41)	17(30)	
Systemic therapy,n(%)			0.696
Yes	36(64)	34(61)	
No	20(36)	22(39)	
Neurologic function status,n(%)			0.897
NO neurologic symptoms:fully active at home/work without assistance	29(51.8)	26(46.4)	
Minor neurologic symptoms:fully active at home/work without assistance	19(33.9)	21(37.5)	
Moderate neurologic symptoms:fully active at home/work but requires assistance	7(12.5)	7(12.5)	
Moderate neurologic symptoms:less than fully active at home/work and requires assistance	1(1.8)	2(3.6)	
Education(>high school v.≤ high school),n(%)			0.980
No formal education	3(5.3)	5(8.9)	
Primary school	26(46.43)	24(42.9)	
Secondary school	14(25)	16(28.5)	
High school	7(12.5)	6(10.7)	
College or associate’s degree	2(3.6)	2(3.6)	
Bachelor’s degree	2(3.6)	2(3.6)	
Master’s degree	2(3.6)	1(1.8)	
Doctoral or professional degree	0	0	

*Abbreviations*: *HA-WBRT+SIB* Hippocampus-avoidance whole-brain radiotherapy with a simultaneous integrated boost, *HA-WBRT* Hippocampus-avoidance whole-brain radiotherapy, *No* Number, *KPS* Karnofsky Performance Status, BMs brain metastases

### Treatment

All patients were diagnosed as multiple brain metastases by contrast-enhanced magnetic resonance imaging (MRI). Among them, 56 patients were treated with HA-WBRT + SIB and another 56 patients received HA-WBRT alone. 112 patients received radiotherapy-planning computed tomography (CT) with 1.5 mm slice thickness in thermoplastic mask, c-pillow and head frame immobilization as well as contrast-enhanced transversal T1-weighted magnetic resonance imaging (MRI). The scanned images were transmitted to Varian’s planning system (Varian Eclipse 8.0 and 15.0 version), and then the CT images were fused with the transversal T1-weighted MRI images and served for the target volume and organ-at-risk delineation.

**Fig. 2:**
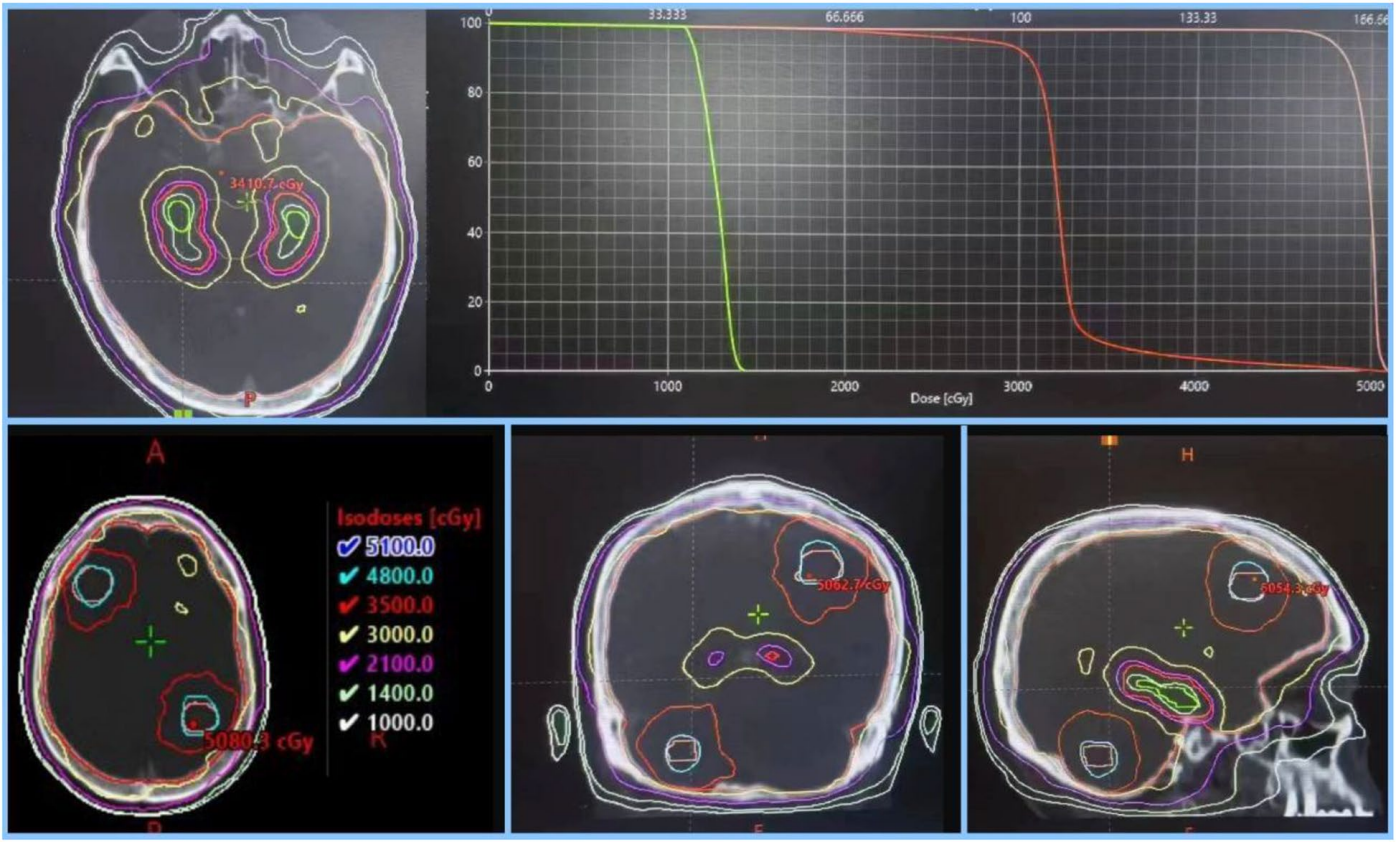
Example of a dose distribution and dose-volume histogram of an HA-WBRT + SIB plan for one patient with 5 brain metastases. Colors indicate the following: green, hippocampi; light pink, PGTVm; orange, PCTV (whole brain + 3 mm); The maximum dose of hippocampus was 14.2 Gy, whereas the whole brain and the metastases received doses of 30 and 48 Gy, respectively. HA-WBRT + SIB, hippocampus-avoidance whole-brain radiation therapy with a simultaneous integrated boost; PGTVm, planning tumor volume of metastases; PCTV, planning tumor volume of the whole brain (clinical target volume)

The whole brain excluding the hippocampus-avoidance region (HAR, a 7 mm 3-dimensional margin around the hippocampus) was defined as the clinical target volume (CTV), and an extension of 3 mm on CTV was defined as planning tumor volume (PCTV) in the observation group. PCTV was given 30 Gy in 12 fractions,per fraction one day,5 fractions per week, maximum dose of the hippocampus (D_max_) ≤ 14 Gy, mean dose (D_mean_) ≤ 9 Gy. The gross tumor volume (GTV_metastases_,i.e. GTV_m_) was contoured on the fused images (CT images and MRI images), GTV_m_ with 2 mm extension formed PGTV_m_,with total dose of 48 Gy in 12 fractions, one fraction every day, 5 fractions one week in the observation group. The whole brain excluding HAR with 3 mm margin was defined as planning tumor volume of the brain (PTV_whole brain_), with the same dose of PCTV of HA-WBRT + SIB in the control group. All plans were adopted volumetric intensity modulated arc therapy (VMAT) and the homogenous dose was prescribed to cover the 95% isodose (Fig. 2). SRT, SRS and SBRT have not been implemented in many places due to unbalanced development in various regions of China. Therefore, the dose escalation to PGTV was used to treat patients with multiple brain metastases (4–15) by linear accelerator (VARIAN CLINAC IX) in this study. Dose constraints of organs at risk are showed in Table 2.

**Table 2:** Dose constraints of Organs-at-Risk

Organs at Risk	Dose constraint
Hippocampus	Dmax≤14Gy
	Dmean≤9Gy
Lens 1-sided	Dmax≤9Gy
Brain stem	Dmax≤40Gy
Inner ear 1-sided	Dmax≤40Gy
Retina, 1-sided	Dmax≤35Gy
Optic nerves, chiasm	Dmax≤35Gy
Optic nerve, 1-sided	Dmax≤35Gy

*Abbreviations*: *Dmax* Maximum dose of Organs-at-Risk, *Dmean* Mean dose of the hippocampus

On the basis of the situation of patients and clinical experience of physicians, 36 patients (HA-WBRT + SIB) and 34 patients (HA-WBRT) underwent more than 2 cycle of systemic therapy, 20 patients (HA-WBRT + SIB) and 22 patients (HA-WBRT) did not undergo systemic therapy.

### Follow‑up and study endpoint

Follow-up was scheduled for examinations in the first month after radiotherapy, including contrast enhanced MRI, clinical examination, and adverse reaction evaluation on the basis of version 4.0 of the Common Terminology Criteria for Adverse Events (CTCAE), and repeated at 3 months interval for the first 2 years, then repeated at 6 months interval in years 3 to 5, and once a year thereafter. The last follow-up was November 2021. Overall survival (OS) ranged from the first day of brain radiotherapy to death or the day on which the patient was last known to be alive in the case of loss to follow-up. Median survival means that only 50% of the individuals can live through this time.Intracerebral control (IC) included complete response (CR), partial response (PR), or stable disease (SD) of brain metastases after radiotherapy. Furthermore, pseudoprogression and radionecrosis related to radiotherapy were excluded from local failures of IC. If the total maximum diameter of treated lesions increased by 20%, or increase the absolute value ≥ 5 mm, or new lesions appeared in the brain, which were considered progressive disease (PD). Intracranial progression-free survival (iPFS) was counted from the time to start radiotherapy until brain metastases progression or death for any reason or the day on which the patient was last known to be alive in the case of loss to follow-up.

Evaluation of treatment-related toxicity included alopecia, radiation dermatitis, encephaledema, headache, emesis, sicchasia, vertigo, fatigue, focal neurologic deficits, epilepsia, neurocognitive dysfunction, and radionecrosis.

### Statistical analysis

Statistical analysis was adopted SPSS 20.0 (IBM Corporation, Chicago, IL, USA) statistical software. To manage the unbalance of potential interference factors, propensity score matching (PSM) was adopted to set up two treatment groups with an even distribution of original characteristics.The propensity score matching analysis was used between HA-WBRT + SIB (the observation group) and HA-WBRT (the control group) to control confounding factors of patients, and performed with a logistic regression that considered the following factors: age, gender, intracranial symptoms, karnofsky performance status, primary tumor, extracranial metastases, maximum size and number of brain metastases. OS, median survival and iPFS were calculated by Kaplan–Meier method. 1-year-IC rates and adverse events were calculated by χ2 test (fisher’s exact test). The baseline characteristics of the patients were counted by χ2 test after matching  *P*<0.05 (two-tailed) of all analysis results were considered statistically significant.

## Results

### Patient characteristics

63 patients treated with HA-WBRT + SIB and 189 patients treated with HA-WBRT were confirmed. All patients were matched through 1:1 propensity score matching analysis, there were 56 patients in each group and were well balanced between them (Table 1). A total of 112 eligible patients with multiple brain metastases were enrolled in the analysis (Fig. 1). Among of them (median age, 58 years old), men were 65 (58%) and women were 47 (42%). Most patients of the two groups were non-small cell lung cancer (NSCLC,71% vs. 29%). The last follow-up date was November 30, 2021, the median follow-up time of the observation group and control group was 11.2 months and 9.8 months, respectively.

### Outcome of overall survival and intracranial progression-free survival

**Fig. 3:**
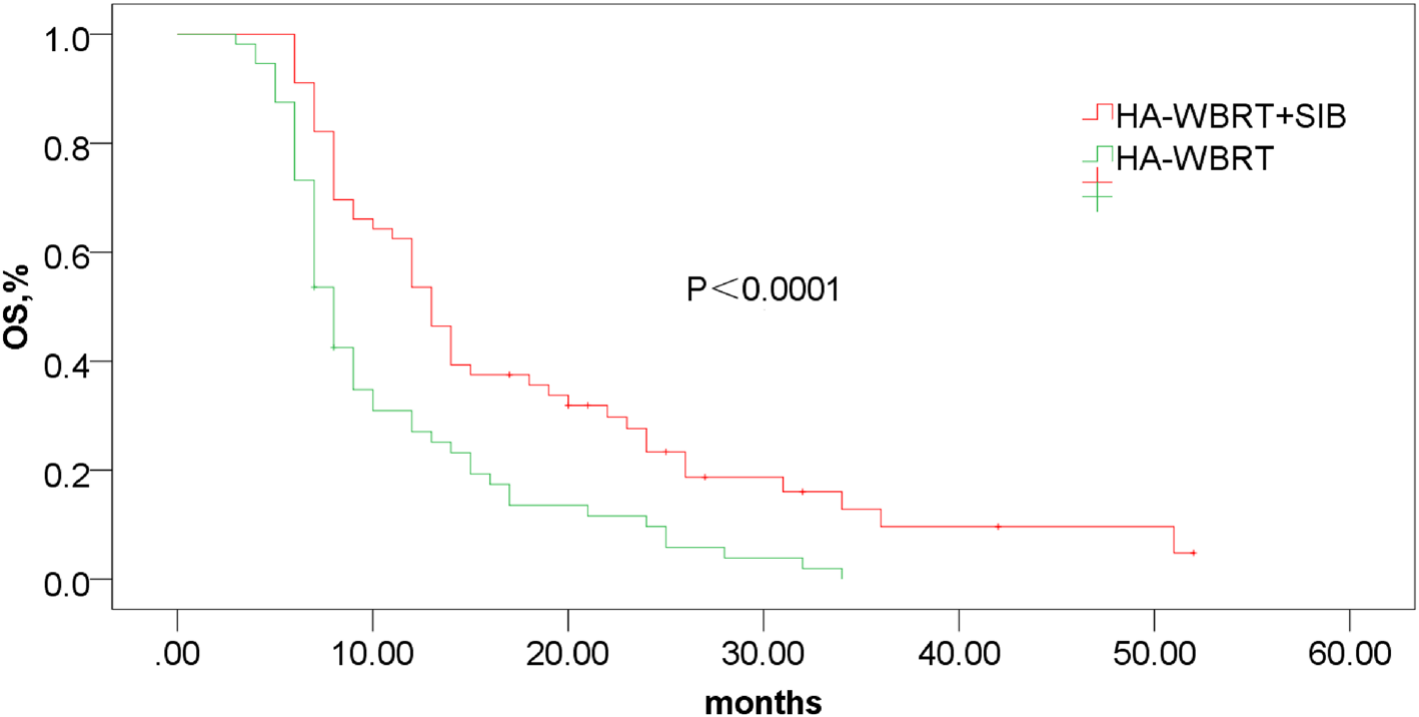
The OS was significantly longer in HA-WBRT + SIB (18.4 vs. 10.9 months, *P*<0.0001). HA-WBRT + SIB,hippocampus-avoidance whole-brain radiation therapy with a simultaneous integrated boost; OS, overall survival; HA-WBRT, hippocampus-avoidance whole-brain radiation therapy

**Fig. 4:**
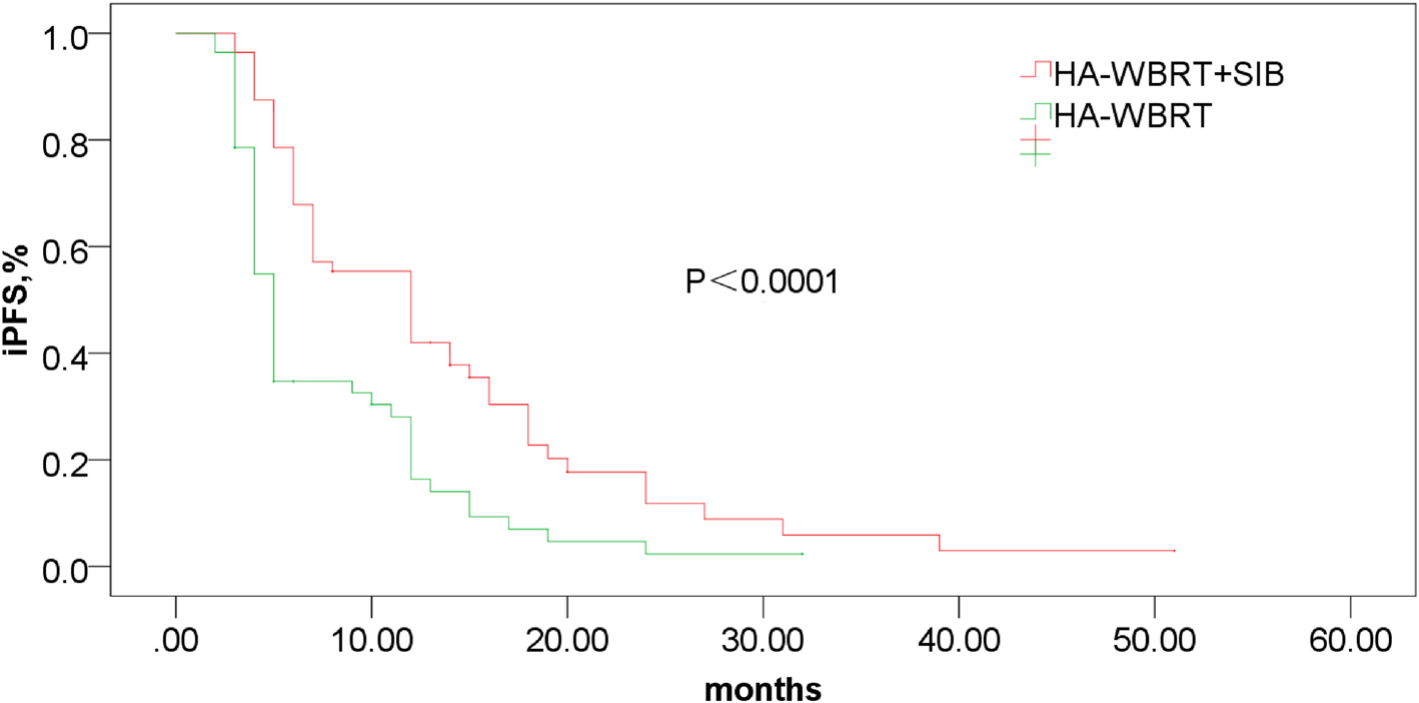
Obviously better iPFS with HA-WBRT + SIB versus HA-WBRT (13.9vs7.8 months, *P*<0.0001). HA-WBRT + SIB, hippocampus-avoidance whole-brain radiation therapy plus a simultaneous integrated boost; iPFS, intracranial progression-free survival; HA-WBRT, hippocampus-avoidance whole-brain radiation therapy

At the end of this study, there were 102 patients of death (48 patients in the observation group, 54 patients in the control group) and 10 survival patients. Among of them, 13 patients of the observation group died in intracranial tumor progression and 30 patients died in extrabrain progression or other reasons (infection, massive hemorrhage, respiratory failure and circulatory failure) and 5 patients died of unknown causes. In the control group, the reason of 21 patients’ death was brain metastases progression, 23 patients died of extracranial progress or other reasons (infection, massive hemorrhage, respiratory failure and circulatory failure), 10 patients of death were unknown. OS improved in the observation group (18.4 vs. 10.9 months, *P*<0.0001; Fig. 3), with a median survival 13 months of the observation group and a median survival 8 months of the control group. Overall iPFS was also longer in the observation group (13.9 vs. 7.8 months, *P*<0.0001; Fig. 4).

#### Intracerebral control

The 1-year IC rate was higher in the observation group (51.8% vs. 21.4%, *P* = 0.002). One month after radiotherapy, there were 442 brain metastases in the observation group, including 127 lesions (28.7%) of complete remission, 172 lesions (38.9%) of partial remission, 61 lesions (13.8%) of stable, and 82 lesions (18.6%) of progress.

There were 429 lesions in the control group, including 56 lesions (13.1%) of complete remission, 125 lesions (29.1%) of partial remission, 64 lesions (14.9%) of stable, and 184 lesions (42.9%) of progress.

### Toxicity of two groups

At the end of follow-up, all adverse events of two groups were assessed according to National Cancer Institute’s CTCAE, version 4.0. Neurocognitive decline 12 months after radiotherapy 3 patients were observed in observation group (3/56,5.4%) and 2 patients were found in the control group (2/56,3.6%), adverse reactions more than grade 2 were 2 patients in the observation group (2/56,3.6%). No significant difference was found in the neurocognitive decline of the two groups (*P* = 0.843), Neurocognitive decline were evaluated according to Mini-mental State Examination (MMSE), including speech ability, orientation, calculation, memory, attention.The total score of MMSE is 30 points, and the evaluation time is about 5-10 min. According to the educational level of patients, the standard of cognitive impairment is divided into general illiteracy ≤ 17 points, primary school education ≤ 20 points, secondary school education ≤ 24 points. Below the standard score, cognitive impairment was considered to exist, which needed to be examined; Severity classification of cognitive impairment: mild MMSE ≥ 21 points; Moderate MMSE 10–20 points; Severe MMSE ≤ 9 points (Table 1 in the supplement). All of them underwent grade 1–2 alopecia (Table 3). The difference between pseudoprogression and radionecrosis related to radiotherapy is detailed in Table 4. Seven hippocampal metastases were found in the two groups (the control group: 4/56,7.1%) and the observation group:3/56,5.4%) after hippocampus-avoidance. The death rate of intracranial progression were 23.2% in HA-WBRT + SIB group and 37.5% in HA-WBRT alone group.

**Table 3:** Adverse events to all patients of the two groups

Adverse Event		Grade					*P* value
		1	2	3	4	5	
alopecia	HA-WBRT+SIB	24	32	0	0	0	0.569
	HA-WBRT	27	29	0	0	0	
radiation dermatitis	HA-WBRT+SIB	31	14	0	0	0	0.792
	HA-WBRT	33	11	0	0	0	
encephaledema	HA-WBRT+SIB	28	21	0	0	0	0.588
	HA-WBRT	26	19	0	0	0	
headache	HA-WBRT+SIB	16	12	0	0	0	0.555
	HA-WBRT	21	9	0	0	0	
emesis	HA-WBRT+SIB	15	13	0	0	0	0.897
	HA-WBRT	16	11	0	0	0	
sicchasia	HA-WBRT+SIB	8	0	0	0	0	0.568
	HA-WBRT	6	0	0	0	0	
vertigo	HA-WBRT+SIB	11	4	0	0	0	0.664
	HA-WBRT	10	2	0	0	0	
fatigue	HA-WBRT+SIB	16	7	0	0	0	0.798
	HA-WBRT	18	5	0	0	0	
focal neurologic deficits	HA-WBRT+SIB	6	1	0	0	0	0.568
	HA-WBRT	5	0	0	0	0	
epilepsia	HA-WBRT+SIB	6	2	0	0	0	0.793
	HA-WBRT	5	1	0	0	0	
neurocognitive dysfunction	HA-WBRT+SIB	2	1	0	0	0	0.843
	HA-WBRT	2	0	0	0	0	
radionecrosis	HA-WBRT+SIB	7	3	2			0.519
	HA-WBRT	6	4	0	0	0	

*Abbreviations*: *HA-WBRT+SIB* Hippocampus-avoidance whole-brain radiotherapy with a simultaneous integrated boost, *HA-WBRT* Hippocampus-avoidance whole-brain radiotherapy

**Table 4:** Pseudoprogression and radionecrosis related to radiotherapy

Project	Pseudoprogression	Radionecrosis
occurrence time	3 months after radiotherapy	More than 6 months after radiotherapy
MRI enhancement	irregular enhancement within the lesion,significant occupancy effect	significant delayed phase enhancement
incidence rate	20-30%, further observation gradually decreases or even disappears	2-18%, progressive and irreversible
18FDG PET-CT	Hypermetabolism	Hypometabolism

*Abbreviations*: *MRI* Magnetic resonance imaging, *18FDG PET-CT* 18F fluorodeoxygluecose position emission tomography imaging

## Discussion

WBRT was considered as the conventional treatment for multiple brain metastases for many years. Although,WBRT + SRS has been recognized the superior choose for the oligometastases in brain,no better OS has been demonstrated in recent years [19–21]. Nevertheless, the best treatment for multiple brain metastases was still controversial. This retrospective research was aimed at assessing the clinical effect and feasibility of HA-WBRT + SIB for multiple brain metastases,and comparing with OS,IC,iPFS for patients treated with HA-WBRT. Altogether HA-WBRT + SIB could improve OS, median survival,IC and iPFS in matched queue (*n* = 112), and higher IC was also associated with longer iPFS. Some studies showed that WBRT with radiation boost was more superior therapy for multiple brain metastases than WBRT alone. Two retrospective researches of Shanghavi et al. [22] and Wang et al. [23] demonstrated that WBRT plus SRS group for multiple brain metastases was better survival than WBRT alone. Shanghavi et al. displayed that the median survival were 16.1, 10.3, and 8.7 months for RPA categories I, II, and III, respectively, showing better survival in WBRT plus SRS group. The other study also showed that survival benefit was found in those patients with low KPS < 70. Wegner et al. suggested that the better survival was found in the cases with SCLC those who underwent WBRT plus SRS than did SRS alone (14 vs. 6 months, *p* = 0.040) [24]. Andrews et al. [8] demonstrated that WBRT plus SRS group had improved general condition and survival compare to the WBRT alone group for patients with one brain metastasis (6.5 vs. 4.9 months; *P* = 0.0393), also showed that 1-year control rate was better in the WBRT plus SRS group (82% vs. 71%, *P* = 0.01). It’s worth noting that merely 24 (7.2%) SCLC cases were contained in the research. These views are similar to this study, but SIB and hippocampus-avoidance were adopted in our study, which could has the biological advantage of dose fractionation and better protect the neurocognitive function.

Some published papers showed that WBRT + SRS did not receive survival benefit in those patients with multiple brain metastases in contrast to SRS alone [25, 26]. So far, SRS has commonly been used to patients with one to three metastases [15, 27]. However, more and more radiotherapy physicians have used SRS for patients with multiple brain metastases instead of WBRT. Many academic researches have demonstrated that SRS has better survival advantage and less toxicity than WBRT In the past 30 years [28]. Jinyu Xue et al. had found that the mean dose of normal brain in SRS was related to the total target volume but not the number of brain metastases [29]. A prospective study (JLGK0901) showed that the survival of patients with 2–4 and 5–10 BMs who had undergone SRS alone were not significant difference, the median OS of these patients was 10.8 months [26, 30, 31].

This study was to compare the OS, iPFS and IC of HA-WBRT and HA-WBRT for patients with multiple brain metastases, to explore the clinical effect and practicability of HA-WBRT + SIB for patients with multiple brain metastases in the context of protecting neurological and neurocognitive function. There were three prospective clinical studies showed that intracranial tumor progression was associated with neurocognitive hypofunction, then decline the quality of life [12, 14, 32]. The 1-year IC rate was obviously higher in the HA-WBRT + SIB group,which increase iPFS. Several randomized researches compared the different dose-fractionation methods of WBRT with conventional dose-fractionation (30 Gy/10f,BED = 39 Gy,α/β = 10), which showed the higher BED for WBRT could not increase survival, but a lower BED could result in worse efficacy [16, 33, 34]. Nevertheless, a lower BED could decline the rate of neurocognitive dysfunction,particularly in case of prophylactic cranial irradiation [35].

Therefore, the lower BED of the whole brain (30 Gy/12f,BED = 37.5 Gy) in the HA-WBRT and HA-WBRT + SIB groups was to decline neurocognitive impairment as far as possible. HA-WBRT is a reliable radiotherapy concept, Because the higher rate of intracranial progression was not associated with the HA. In this study, four hippocampal metastases were found in HA-WBRT (4/56,7.1%), 3 patients were found single metastatic lesion in unilateral hippocampus, only one patient was found a lesion in bilateral hippocampus, respectively. Three hippocampal metastases were found in HA-WBRT + SIB (3/56,5.4%), 3 patients were found single metastatic lesion in unilateral hippocampus. The data analysed showed that all brain metastases been treated are far from the hippocampus, which led to the lower dose of hippocampus and may be related to hippocampal metastasis several researches showed the similar rates of new hippocampal metastases after HA-WBRT [36–38, 40].

In this study, the data in the HA-WBRT + SIB group demonstrated that the 4–15 brain metastases could improve 1-year rates, OS and iPFS, which were the same as the researches [39, 40]. Dose limit of hippocampus (Dmax ≤ 14 Gy) in the two groups were not found significant difference, we selected the maximum limit of hippocampus in the two groups owing to the dose of hippocampus being related to predict neurocognitive dysfunction [41, 42], which was slightly different from Phase III Trial NRG Oncology CC001 [17]. There are three limitations in our study, Firstly, There was no SRS control group in our study, therefore we could not compare with SRS group. Then, there were not univariate and multivariate analyses of overall survival.Finally, the material of retrospective study maybe bias the outcomes and did not take memantine during HA-WBRT.

## Conclusions

In this study, HA-WBRT + SIB resulted in better OS, median survival, IC, iPFS, an acceptable toxicity, and a potential way of declining neurocognitive adverse events, which may be a better treatment for patients with multiple brain metastases.

## Data Availability

The original contributions presented in the study are included in the article/supplementary material.Further inquiries can be directed to the corresponding author.

## Abbreviations

WBRT: Whole Brain Radiotherapy
HA-WBRT: Hippocampus-Avoidance Whole-Brain Radiotherapy
HA-WBRT+SIB: Hippocampus-Avoidance Whole-Brain Radiotherapy plus Simultaneous Integrated Boost
OS: Overall Survival
IC: Intracranial Sontrol
iPFS: Intracranial Progression-Free Survival
BMs: Brain Metastases
LC: Local Control
CT: Computed Tomography
MRI: Magnetic Resonance Imaging
KPS: Karnofsky Performance Status
GTV: The Gross Tumor Volume
CTV: Clinical Target Volume
PGTV: The planning gross tumor volume
PCTV: The Planning Clinical Target Volume
CTCAE: The Common Terminology Criteria for Adverse Events
NSCLC: Non Small Cell Lung Cancer
PSM: Propensity Score Matching
CR: Complete Response
PR: Partial Response
SD: Stable Disease
PD: Progressive Disease
